# YAP-Driven Malignant Reprogramming of Epithelial Stem Cells at Single Cell Resolution

**DOI:** 10.21203/rs.3.rs-3426301/v1

**Published:** 2023-10-27

**Authors:** J. Silvio Gutkind, Farhoud Faraji, Sydney Ramirez, Lauren Clubb, Kuniaki Sato, Paola Anguiano Quiroz, William Galloway, Zbigniew Mikulski, Thomas Hoang, Kate Medetgul-Ernar, Pauline Marangoni, Kyle Jones, Adam Officer, Alfredo Molinolo, Kenneth Kim, Kanako Sakaguchi, Joseph Califano, Quinton Smith, Ophir Klein, Pablo Tamayo

**Affiliations:** University of California, San Diego; University of California San Diego Health Department of Otolaryngology-Head and Neck Surgery and Moores Cancer Center; La Jolla Institute of Immunology; University of California San Diego Health Moores Cancer Center; University of California San Diego Health Moores Cancer Center; University of California San Diego Health Moores Cancer Center; University of California Irvine Department of Chemical and Biomolecular Engineering; La Jolla Institute of Immunology; University of California San Diego Health Moores Cancer Center; University of California San Diego Health Moores Cancer Center; Craniofacial Biology and Department of Orofacial Sciences, University of California, San Francisco; University of California San Francisco (UCSF); University of California San Diego Health Moores Cancer Center; University of California San Diego Health Moores Cancer Center; La Jolla Institute of Immunology; IDEXX Laboratories KK; University of California San Diego; University of California Irvine Department of Chemical and Biomolecular Engineering; Cedars-Sinai Medical Center, Los Angeles; University of California, San Diego

**Keywords:** Tumor initiation, Tumor initiating cell, Cancer stem cell, Squamous cell carcinoma, Oral cancer, HNSCC, Hippo pathway, YAP, HPV

## Abstract

Tumor initiation represents the first step in tumorigenesis during which normal progenitor cells undergo cell fate transition to cancer. Capturing this process as it occurs *in vivo*, however, remains elusive. Here we employ cell tracing approaches with spatiotemporally controlled oncogene activation and tumor suppressor inhibition to unveil the processes underlying oral epithelial progenitor cell reprogramming into cancer stem cells (CSCs) at single cell resolution. This revealed the rapid emergence of a distinct stem-like cell state, defined by aberrant proliferative, hypoxic, squamous differentiation, and partial epithelial to mesenchymal (pEMT) invasive gene programs. Interestingly, CSCs harbor limited cell autonomous invasive capacity, but instead recruit myeloid cells to remodel the basement membrane and ultimately initiate tumor invasion. CSC transcriptional programs are conserved in human carcinomas and associated with poor patient survival. These findings illuminate the process of cancer initiation at single cell resolution, thus identifying candidate targets for early cancer detection and prevention.

## INTRODUCTION

Adult stem cells play a central role in tissue homeostasis by balancing self-renewal and differentiation.^1^ However, cells with self-renewal capacity may also accumulate and propagate oncogenic genomic alterations, ultimately leading to carcinogenesis.^2,3^ Stem cells thus possess intrinsic tumor suppressive mechanisms to guard against inappropriate oncogene activation, including terminal differentiation,^4^ oncogene-induced senescence,^5^ and apoptosis.^6^ Current models of carcinogenesis posit that tumor initiation requires the inactivation of intrinsic tumor suppressive mechanisms concomitant with oncogene activation.^7^ These insights have been supported by recent genome-wide cancer sequencing efforts that have cataloged candidate genomic alterations underlying most human malignancies.^8^ However, these studies in established, often advanced tumors are confounded by cellular and mutational heterogeneity and thus cannot directly identify cancer-initiating cells, often referred to as cancer stem cells (CSCs), or discriminate between alterations driving tumor initiation from those promoting tumor progression. As such, the underlying molecular mechanisms mediating malignant reprogramming of normal progenitor cells into CSCs remains poorly understood.

Stem cells in the oral mucosa reside in the basal layer of the stratified squamous epithelium, and consist of a single pool of self-renewing oral epithelial progenitor cells (OEPCs).^9^ This single progenitor cell population renders the oral epithelium an ideal system to elucidate mechanisms underlying malignant reprogramming,^10^ unlike other tissue systems such as the skin or gut, in which elegant studies have revealed multiple unique stem cell pools that play distinct roles in cancer initiation.^11,12^ Head and neck squamous cell carcinoma (HNSC) represents the most common malignancy arising from the upper aerodigestive epithelia.^13^ Extensive molecular characterization of HNSC has revealed that while each individual tumor exhibits alterations in a large number of genes, these alterations converge to impact a finite set of oncogenic molecular pathways.^14^ HNSC is characterized by near universal loss-of-function of *TP53* and *CDKN2A* tumor suppressors by genomic alteration or human papillomavirus (HPV)-mediated inhibition.^14^ Notably, alterations in *FAT1*, observed in nearly 30% of HNSC,^14^ disrupt Hippo pathway signaling and result in unrestrained activation of the transcriptional co-activator YAP.^15^ We now combine knowledge of the landscape of oncogenic pathway alterations in HNSC with genetically engineered animal models, lineage tracing, and single cell transcriptomics to unveil the underpinnings of cancer initiation *in vivo*. Here, we found that in the context of *TP53* and *CDKN2A* suppression, YAP activation rapidly reprograms OEPCs into CSCs, providing an opportunity to illuminate the process of cancer initiation at single cell resolution.

## RESULTS

### YAP activation and E6-E7 expression is sufficient to induce rapid tumor initiation

**Figure 1:**
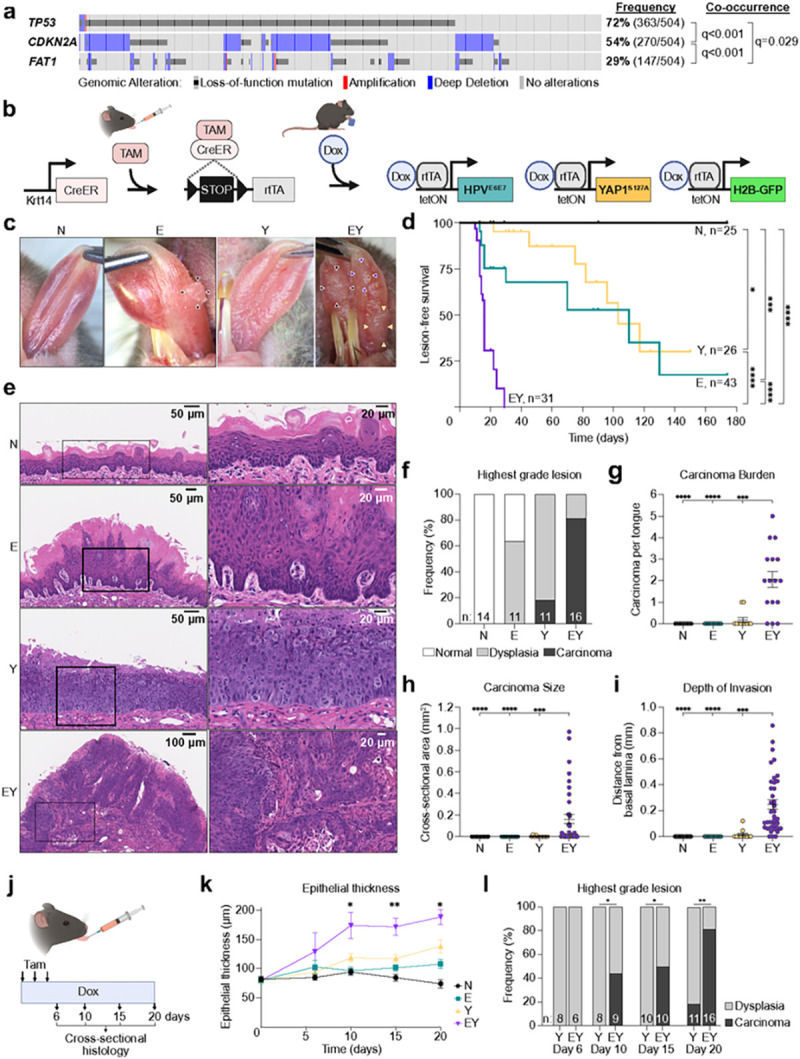
YAP and E6-E7 activation is sufficient to induce rapid tumor initiation in OEPCs (a) Frequency and co-occurrence of *TP53, CDKN2A,* and *FAT1* genomic alterations in the The Cancer Genome Atlas (TCGA) Head and Neck Squamous Carcinoma (HNSC) cohort (n=504). (b) Schematic depicting spatiotemporally controlled activation of YAP and HPV oncogenic pathways in oral epithelial progenitor cells. A tissue-specific inducible Cre recombinase (CreERT) expressed in *Krt14*-expressing cells is activated by intralingual injection of tamoxifen (Tam), resulting in recombination of the Lox_p_-STOP-Lox_p_ cassette (LSL) and expression of the rtTA transgene. Feeding tamoxifen-treated mice doxycycline chow (Dox) activates transcription of the tetracycline-inducible transgenes *HPV16*^*E6-E7*^*, YAP*^*S127A*^, and *H2B-GFP* reporter. (c) Representative images of gross tongue lesions 20 days after transgene induction. (d) Kaplan-Meier plot showing the kinetics of tongue lesion formation upon transgene induction. (e) Hematoxylin and eosin (H&E) stained tongue epithelia sections demonstrating epithelial changes 20 days after transgene activation. (f) Histopathologic evaluation and scoring of mouse tongue epithelia; n indicates number of tongues examined for each transgenic condition. Dysplasia = any dysplastic changes present. Carcinoma = infiltrative lesions invading beyond the basement membrane. (g) Number of infiltrative carcinomatous lesions per examined tongue. Panels G and H: n_Y_=2, n_EY_=34 carcinomata. (h) Cross-sectional area of infiltrative carcinomatous lesions. (i) Depth of carcinoma invasion as measured by the longest plumb line orthogonal to the tangent of the nearest intact basement membrane. (j) Schematic showing pulse-chase strategy of transgene induction and time points for histological analysis of tongue epithelia. (k) Longitudinal measurement of tongue epithelial thickness. n=3–7 mice per condition per time point. (l) Highest grade lesion identified per tongue for the carcinogenic conditions Y and EY. Panel d was analyzed by Bonferroni-corrected Mantel-Cox log-rank test. Panels g-i, and k were analyzed by ANOVA with Tukey correction for multiple comparisons. Panel l was analyzed by two-sided Fisher’s exact test. For all panels with asterisks denoting significance: **p<0.01, ***p<0.001, ****p<0.0001.

Tumor initiation represents the crucial first step in tumorigenesis during which normal progenitor cells undergo cell fate transition to cancer. To investigate this process, we developed genetically engineered murine systems focusing on prevalent and co-occuring genomic alterations in HNSC (Fig. 1a). We employed conditional expression of the HPV16 E6-E7 oncogenes to concomitantly inhibit the *TP53* and *CDKN2A* tumor suppressors.^16^ Given the frequency of *FAT1* mutations and widespread YAP nuclear expression in HNSC,^17^ we investigated the effects of YAP activation in conjunction with E6-E7 by expression of the *YAP1*^*S127A*^ allele. Keratin 14 (KRT14) is expressed in the basal layer of oral epithelia and marks OEPCs, which may represent the cell of origin for HNSC.^18^ We employed a tamoxifen-inducible Cre-recombinase (CreERT) driven by the *Krt14* promoter to target genomic alterations to KRT14^+^ OEPCs.^19^ We bred mice bearing E6-E7 (“E”), *YAP1*^*S127A*^ (“Y”), or both transgenes (“EY”). Littermates lacking these transgenes but possessing *Krt14-CreERT, LSL-rtTA* regulatory transgenes, and the H2B-GFP reporter were used as normal controls (“N”). Local administration of tamoxifen activated CreERT-mediated recombination of a floxed STOP cassette (LSL) and enabled transcription of the reverse tetracycline-controlled transactivator (*rtTA*) in KRT14^+^ OEPCs (Fig. 1b). Administration of doxycycline chow then induced expression of the tetracycline response element-regulated *HPV16*^*E6 − E7*^*, YAP1*^*S127A*^, and *H2B-GFP* transgenes (Extended Data Fig. 1a-d).^16,20,21^

Serial examination of mouse tongues identified macroscopic lesions as early as 8 days after transgene induction. Median lesion-free survival was dramatically different across genotypes (Fig. 1c–d). By 20 days, the majority (65%) of EY mice bore at least two lesions, while few Y and no E or N mice bore any lesions (Extended Data Fig. 1e). Evaluation of hematoxylin and eosin (H&E) staining and pan-cytokeratin immunohistochemistry (IHC, Extended Data Fig. 1f) showed at least one invasive carcinoma in 81% of EY mice, compared to 18% of Y mice and no E or N mice (Fig. 1e,f). Most carcinoma-bearing EY mice had multiple independent carcinomas, which were also larger and more deeply invasive than those of Y mice (Fig. 1g–i). We next investigated tumor initiation at higher temporal resolution using a pulse-chase strategy. At day 10, we observed a marked increase in EY epithelial thickness compared to other epithelia (Fig. 1j,k). Invasive carcinoma occurred in 44% of EY mice by 10 days, while no carcinoma in Y epithelia were observed until day 20 (Fig. 1l). These findings show that in KRT14^+^ OEPCs, unrestrained YAP activation in the context of E6-E7 expression is sufficient to induce oral carcinoma with high penetrance and rapid kinetics.

### Invasive carcinoma is preceded by the expansion of a stem-like cell population

**Figure 2:**
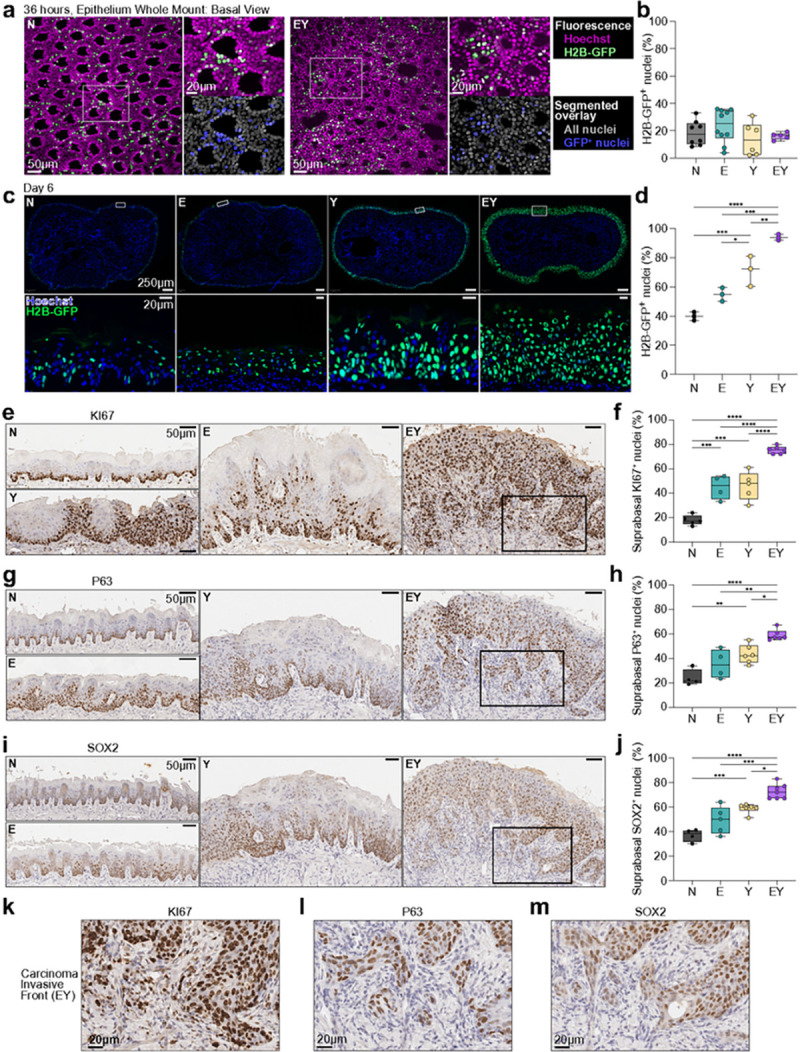
Invasive carcinoma is preceded by the expansion of a stem-like cell population (a-d) Lineage tracing by fluorescent microscopy using the *H2B-GFP* reporter transgene to track and quantify GFP^+^ nuclei. (a) Basal cells in tongue epithelial whole mounts 36 hours after transgene induction. (b) Percent basal H2B-GFP^+^ nuclei in a. (c) Representative axial tongue sections of H2B-GFP^+^ nuclei 6 days after transgene induction. (d) Percent H2B-GFP^+^ nuclei in c. (e-j) Representative IHC images 20 days after transgene induction of (e) KI67^+^ nuclei, (g) P63^+^ nuclei, (i) SOX2^+^ nuclei. Percent of positively stained nuclei in the suprabasal epidermal strata for: (f) KI67, (h) P63, (j) SOX2. (k-l) Magnified images of cells at the invasive front demonstrating (k) KI67^+^ nuclei in e, (l) P63^+^ nuclei in g, (m) SOX2^+^ nuclei from i. Panels b, d, f, h, and j were analyzed by ANOVA with Tukey correction for multiple comparisons. For all panels with asterisks denoting significance: *p<0.05, **p<0.01, ***p<0.001, ****p<0.0001.

We investigated the early processes of tumor initiation employing *H2B-GFP* for lineage tracing of transgene-activated cells. In epithelial whole mounts 36 hours after a single dose of intralingual tamoxifen, we observed a similar distribution of H2B-GFP^+^ basal cells across transgenic conditions (Fig. 2a,b). By 6 days, H2B-GFP^+^ cells had expanded throughout the full thickness of EY epithelia (Fig. 2c,d). While isolated mitotically active cells (KI67^+^ staining) remained restricted to the basal layer in normal (N) epithelia, the expression of E, Y, or EY transgenes resulted in extension of KI67^+^ cells beyond the basal layer (Fig. 2e,f, Extended Data Fig. 2a,c). We next evaluated the expression of P63 and SOX2, transcription factors that regulate epithelial morphogenesis and OEPC maintenance,^22,23^ and the transmembrane protein ITGA6, a component of the hemidesmosome.^24,25^ In normal epithelia, P63 and SOX2 expression was restricted to the basal layer, and ITGA6 to basal cells in contact with the basement membrane. Expression of E, Y, or EY led to an increase in P63^+^ epithelial cells. YAP activation resulted in ectopic extension of P63^+^ cells into suprabasal epithelial layers. Concomitant YAP and E6-E7 expression resulted in expansion of the P63^+^ compartment to occupy the full thickness of the oral epithelia (Fig. 2g,h, Extended Data Fig. 2b,d). Similarly, EY expression drove the expansion of SOX2^+^ cells into all suprabasal layers (Fig. 2i,j). While E6-E7 expression alone did not alter ITGA6 compartmentalization, Y and EY expression diminished ITGA6 basement membrane localization and resulted in diffuse low (Y) to high (EY) ITGA6 expression throughout suprabasal strata (Extended Data Fig. 2b,e). Strikingly, in EY tumors, most cells at the invasive front were KI67^+^/TP63^+^/SOX2^+^ (Fig. 2k–m). Taken together, these findings suggest that YAP activation, together with E6-E7-mediated tumor suppressor inhibition, results in the rapid expansion of a proliferating stem cell-like population at the pre-invasive stage.

### Transcriptional programs underlying OEPC reprogramming: YAP promotes mTOR signaling

**Figure 3:**
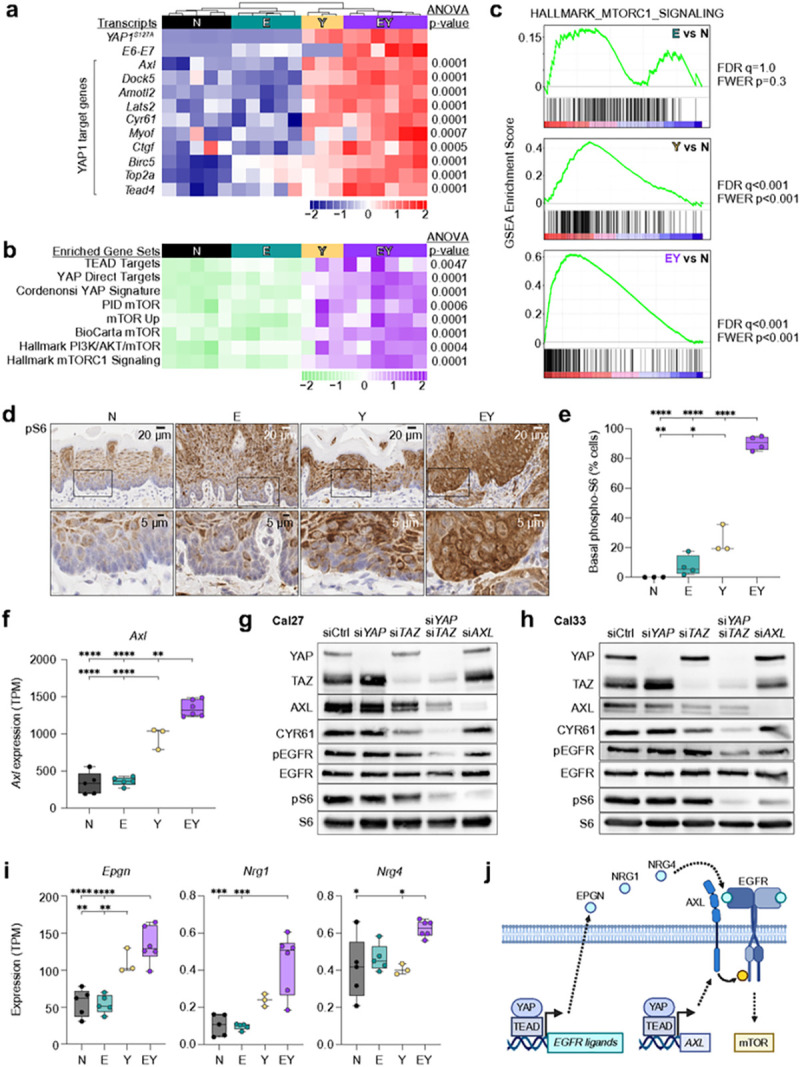
YAP promotes mTOR signaling (a) YAP target gene expression in transgenic epithelia transcriptomes. (b) GSEA for gene sets associated with TEAD- and YAP-target gene expression, and mTOR activation. For gene set details, please see Supplementary Table 1. (c) GSEA enrichment plots for the HALLMARK_MTORC1_SIGNALING gene set for E-, Y-, and EY-specific differentially expressed genes. Right: GSEA normalized enrichment scores and statistics for E-, Y-, and EY-specific differentially expressed genes. (d) IHC staining and (e) quantification of phospho-S6-positive cells in basal epithelial cells. (f) YAP-target gene *Axl* expression. (g,h) Western blot showing effect of siRNA-mediated knockdown of YAP, TAZ, and AXL on YAP target gene abundance and the abundance and phosphorylation of EGFR and S6 ribosomal protein in (g) Cal27 and (h) Cal33 cells. (i) Expression of EGFR ligands *Epgn, Nrg1*, in *Nrg4* in tongue epithelia. (j) Proposed model for YAP-mediated transcriptional activation of mTORC1 via AXL and EGFR ligands. Panels a, b, e, f, and i were analyzed by ANOVA with Tukey correction for multiple comparisons. For all panels with asterisks denoting significance: *p<0.05, **p<0.01, ***p<0.001, ****p<0.0001.

**Figure 4:**
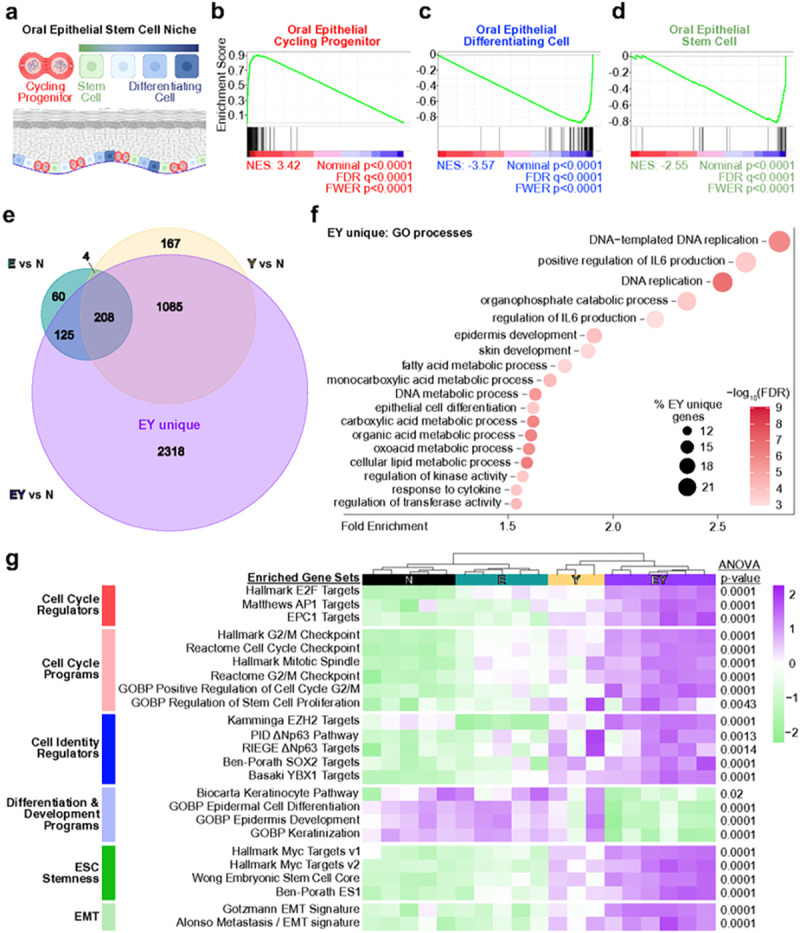
Oncogenic transcriptional reprogramming defines YAP and E6-E7 activated epithelia (a) Model of the basal layer oral epithelial stem cell niche depicting physiologic cell states including stem cells, cycling progenitor cells, and differentiating cells. Adapted from Jones et al.^9^ (b-d) GSEA analysis enrichment plots of EY vs N mouse tongue epithelium differentially expressed genes for (b) cycling progenitor cell (G1/S, G2/M), (c) differentiating cell, and (d) stem cell oral epithelial cell programs. (e) Venn analysis of shared and unique differentially expressed genes for each transgenic condition versus control (N). ‘EY unique’ denotes the 2,421 genes uniquely dysregulated in EY. (f) Enriched cellular processes among EY unique DEGs by Gene Ontology. (g) Single sample GSEA for all transgenic conditions for gene signatures related to cell cycle, differentiation, stemness, and EMT. For gene set details, please see Supplementary Table 1. Each row in panel g was analyzed by ANOVA with Tukey correction for multiple comparisons: *p<0.05, **p<0.01, ***p<0.001, ****p<0.0001.

We performed RNA sequencing (RNAseq) of microdissected tongue epithelia to evaluate transcriptional changes occurring upon tumor initiation (Extended Data Fig. 3a). We selected 15 days post-transgene induction as the normal to carcinoma transition point since half of EY mice bore invasive carcinoma at this time point. Principal component analysis showed clear distinctions between EY, Y, E, and N samples (Extended Data Fig. 3b). As expected, upregulation of YAP target genes^26^ and YAP signatures by gene set enrichment analysis (GSEA)^27^ were observed in YAP-expressing conditions (Fig. 3a,b, Extended Data Fig. 3c,d, Supplementary Table 1), including cell cycle, cell identity, differentiation, and stemness programs (see below Fig. 4g).

Unexpectedly, among multiple transcriptional signatures, Y and EY mice also showed enrichment for gene sets for mTOR pathway activation, a commonly activated signaling mechanism in HNSC^28^ (Fig. 3b,c and Extended Data Fig. 3e). Consistently, IHC of epithelia from EY and Y mice showed a pronounced increase in phospho-S6 (pS6) ribosomal protein levels, a downstream marker of mTOR activity^29^ (Fig. 3d,e). These findings raised the possibility that YAP may drive mTOR activity. We noted that *Axl*, a YAP target gene, is upregulated in epithelia of Y and EY mice (Fig. 3f), potentially representing a mechanistic candidate linking YAP activation to mTOR signaling. We performed siRNA-mediated knockdown of YAP and/or its paralog *TAZ (WWTR1)* in human HNSC cells (Cal27 and Cal33) to test if YAP indeed regulates mTOR activity. Consistent with reports that YAP and TAZ serve mutually compensatory functions,^30^ combined knockdown of *YAP* and *TAZ* was required to diminish expression of the YAP target *CYR61* and mTOR activity, based on pS6 abundance (Fig. 3g,h). Intriguingly, combined knockdown also resulted in diminished pEGFR but not total EGFR, potentially related to decreased expression of YAP-regulated EGFR ligands, including *Epgn, Nrg1, and Nrg4* (Fig. 3i), suggesting that YAP initiates multiple transcriptional mechanisms resulting in mTOR activation (Fig. 3j).

### Oncogenic transcriptional reprogramming defines YAP and E6-E7 activated epithelia

We next extended our global pathway analysis to OEPC gene programs. Recent characterization of normal murine oral epithelia at single cell resolution showed that the basal layer consists of stem, cycling progenitor, and differentiating cells (Fig. 4a).^9^ Using signatures of these physiologic cell states, we evaluated the effects of transgene expression in bulk oral epithelia. We observed pronounced enrichment for the cycling progenitor signature and a dramatic depletion of the differentiation signature, suggesting that EY expression promotes oral epithelial cell cycle progression concomitant with differentiation arrest and depletion of the normal stem cell program (Fig. 4b,d and Extended Data Fig. 4a). At the gene level, differentially expressed gene (DEG) analysis showed EY-mediated upregulation of OEPC stemness factors (*Ybx1, Procr, Ezh2, Suz12, Eed*) and downregulation of differentiation (*Grhl1, Ovol1, Krt4, Krt13, Ivl, Lor, Krtdap,* late cornified envelope genes) and apicobasal polarity factors (*Pard3, Pard3b, Vangl2, Camsap3*). Notably, several basal progenitor state factors (*Hoxc13, Pax9, Gli2, Krt15*) displayed paradoxical downregulation (Extended Data Fig. 4b), indicating that EY-driven transcriptional changes do not reflect a physiologic OEPC cell state, but rather constitute a unique cellular state related to tumor initiation.

To gain a global view of transcriptional programs, DEGs in each condition were compared to normal. From E to Y to EY, a progressively larger number of DEGs was observed (Supplementary Table 2). Venn analysis revealed 2318 genes differentially expressed solely in EY mice (Fig. 4e, Supplementary Table 3). Gene ontology (GO)^31,32^ and GSEA of Molecular Signatures Database (MSigDB) Hallmark pathways^33,34^ identified enrichment among these ‘EY-unique’ DEGs for processes underlying cell proliferation, epithelial cell development and identity, and acute phase inflammatory responses (Fig. 4f and Extended Data Fig. 4c). Further analysis revealed that distinguishing features of EY transcriptomes included activation of cell cycle and stemness programs, inhibition of differentiation, and activation of epithelial to mesenchymal transition (EMT, Fig. 4g). These findings suggest that beyond cell cycle activation, the OEPC to CSC transition involves differentiation arrest, activation of stemness, and EMT-induced epithelial plasticity.

### Activation of CSC programs in nascent carcinoma at single cell resolution

**Figure 5:**
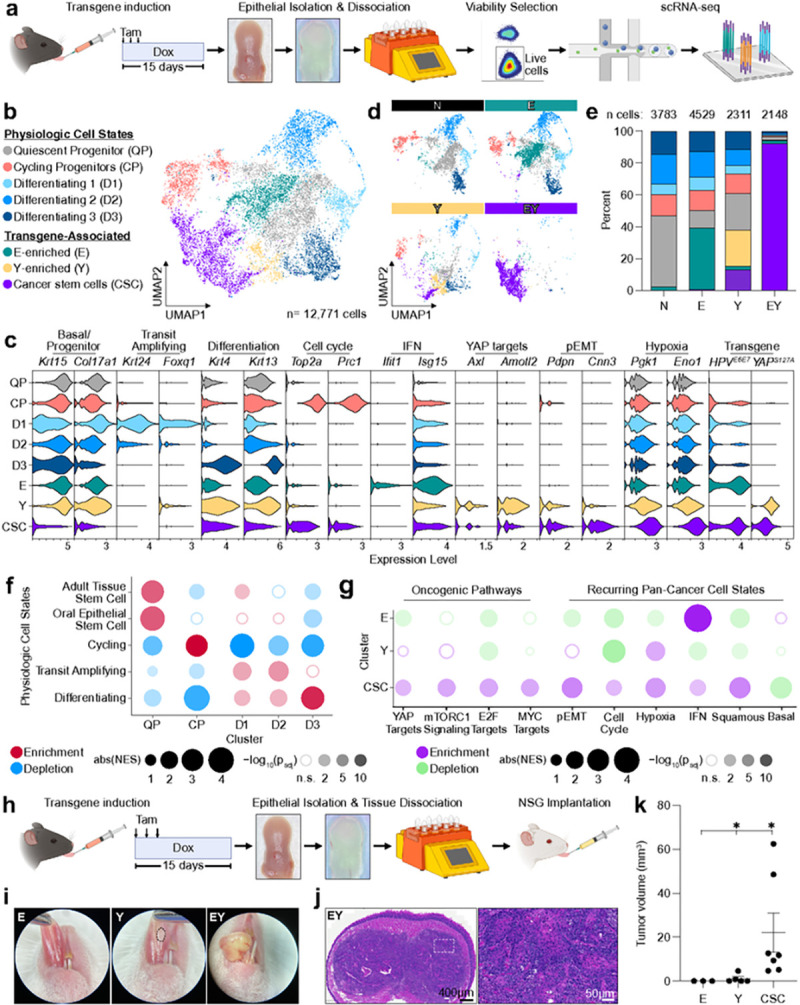
Activation of CSC programs in nascent carcinoma at single cell resolution (a) Experimental approach for single cell transcriptome profiling of tongue epithelia 15 days after transgene induction. (b) UMAP of epithelial cell states (clusters) identified by scRNAseq classified into physiologic and transgene-associated cell states, n=12,771 epithelial cells. (c) Relative expression of representative cell state associated genes by cluster. (d) Individual UMAPs by transgenic condition demonstrating contributions to overall epithelial cell UMAP shown in B. n=2 mouse tongue epithelia per transgenic condition. (e) Distribution of cell states in tongue epithelial cells from each transgenic condition. n = total cells per transgenic condition. (f-g)GSEA of (f) physiologic cell states across epithelial cell clusters and (g) molecular signatures and recurring cancer cell states across the transgene-enriched clusters. Dot color indicates enrichment (red in f, purple in g) or depletion (blue in f, green in g). Dot size encodes the absolute value (abs) of the normalized enrichment score (NES). Dot opacity represents −log_10_ of the adjusted p-value (p_adj_); dots hollow if p_adj_>0.05. For gene set details, please see Supplementary Table 1. (h) Experimental approach for orthotopic implantation of transgene-induced epithelial cell suspensions. (i) Tumor volumes 10 days after implantation. *p<0.05. (j) Representative photographs of mouse tongues 10 days after implantation. (k) H&E stained sections of a representative tongue with an implanted EY cell-derived tumor 10 days after EY cell implantation. ANOVA with Tukey correction for multiple comparisons: *p<0.05.

Our transcriptional analyses of bulk epithelia shed insight into the processes occurring in cells undergoing malignant conversion. However, the cellular heterogeneity of the pre-malignant microenvironment precludes elucidation of tumor initiating transcriptional programs specifically in CSCs. We thus performed single cell RNAseq (scRNAseq) of oral epithelia at the same time point as bulk RNAseq (Fig. 5a). In total, 12,771 epithelial cells were identified across 8 clusters, which could broadly be divided into defined physiologic cell states^9^ (see above, Fig. 4a) and transgene-associated cell states (Fig. 5b). As a first step in assigning cluster identities, we performed GSEA comparing epithelial clusters to published transcriptional signatures for normal oral epithelial cell states.^9,35–37^ We identified physiologic clusters with Quiescent Progenitors (‘QP’, 2773 cells), Cycling Progenitors (‘CP’, 1376 cells), and Differentiating (‘D1’, 1066 cells, ‘D2’, 1674 cells, ‘D3’, 1422 cells) cell phenotypes. Differentiating cells exhibited a continuum of maturation states with progressively increasing enrichment for the differentiating cell signature from D1 to D3 (Fig. 5f and Extended Data Fig. 5d). At the gene level, Quiescent Progenitor cells displayed enrichment of basal/progenitor markers (*Krt15, Col17a1, Itgb4, Itga6*), factors required for stem cell maintenance (*Trp63, Sox2, Zfp36, Procr, Snai2*), enrichment of Notch pathway members (*Hes1, Jag2*), and antiproliferative factors (*Btg2, Gas1*). Cycling Progenitor cells showed enrichment for factors required for G1/S and G2/M transition (*Foxm1, Pcna, Ccna2, Ccnb1, Cdc25b, Cdk1*), mitosis (*Top2a, Prc1, Mki67, Cenpf, Tpx2*), and showed depletion of differentiation markers (*Krtdap, Krt4, Krt13*). D1 cells were enriched for markers of lineage commitment (*Krt24*),^38^ factors required for transit amplifying cell proliferation (*Foxq1*),^37,39^ and factors required for cell fate determination (*Hoxc13, Pax9*).^40,41^ D2 and D3 cells showed increasing expression of terminal differentiation markers (*Krt4, Krt13, Krtdap, Fabp5*) (Fig. 5c, Extended Data Fig. 5a, and Supplementary Table 4). These findings support that the identities assigned to our clusters are consistent with defined normal oral epithelial cell states.

E, Y, and EY transgene expression resulted in the emergence of three clusters not observed in normal epithelia (Fig. 5d,e and Extended Data Fig. 5b,c). The majority of E-enriched cluster cells displayed high expression of E6-E7 and interferon-stimulated genes known to be upregulated by E6-E7 (*Cxcl10, Ifit1, Isg15, Ifi27l2a, Ifitm3*).^42,43^ The YAP-enriched cluster showed high expression of YAP1^S127A^ and YAP target genes (*Axl, Amotl2, Lats2, Cyr61*). Finally, the emergence of a unique cluster resembling cancer stem cells (‘CSC’, 2,324 cells) was observed in EY epithelia. CSCs expressed both *E6-E7* and *YAP1*^*S127A*^ transgenes, and displayed high expression of pEMT (*Cnn3, Lgals1, Anxa3, Ndrg1, Pdpn*) and hypoxia (*Pgk1, Eno1, Ldha, Aldoa, Tpi1*) transcripts (Fig. 5c).^44,45^ The CSC cluster was markedly enriched for YAP, mTORC1, E2F, and MYC-driven transcriptional programs, suggesting that these key oncogenic pathways are exclusively activated in CSCs. Indeed, the CSCs were strongly enriched for transcriptional modules defining pan-cancer cell states^44^ including pEMT and hypoxia as described above, plus cell cycle, interferon response, and squamous differentiation (Fig. 5g and Extended Data Fig. 5e). Aligned with this possibility, the pEMT marker podoplanin (PDPN) is highly expressed in CSCs invading the basement membrane (Extended Data Fig. 5f).

To test tumor initiating capacity, we implanted cell suspensions generated from E, Y, and EY transgene induced epithelia into the tongues of NOD-SCID-gamma (NSG) immune compromised mice (Fig. 5h). Large tumors formed in all mice implanted with EY-induced cells, compared to only small tumors in fewer mice implanted with Y-induced cells, and no tumors in mice implanted with E-induced cells (Fig. 5i–k). Collectively, these findings demonstrate that combined YAP activation and E6-E7 expression endows OEPCs with an oncogenic transcriptional phenotype, and results in efficient OEPC reprogramming into CSCs.

### CSCs co-opt collagenase-expressing G-MDSCs to facilitate tumor invasion

**Figure 6:**
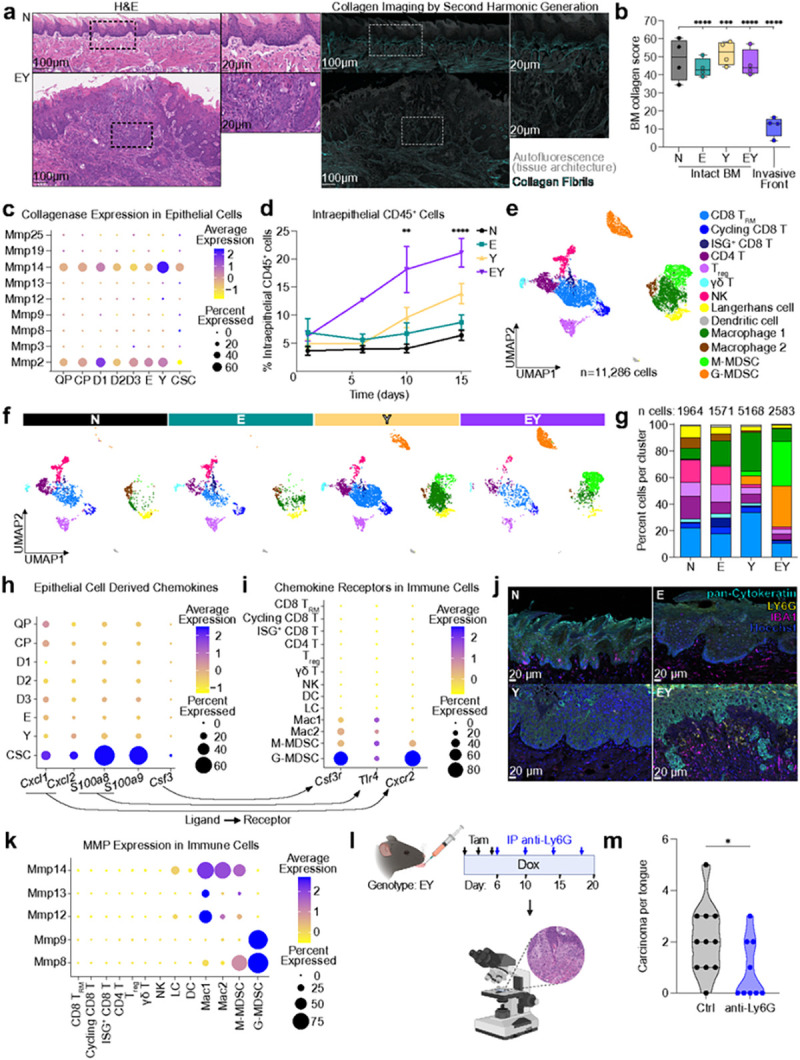
CSCs co-opt collagenase-expressing G-MDSCs to facilitate tumor invasion (a) Representative H&E-stained sections (left) and collagen imaging by second harmonic generation (right) in control epithelia (N) and an infiltrative carcinoma lesion (EY) 20 days after transgene induction. (b) Fluorescent intensity of second harmonic generation signal at intact basement membrane of transgenic tongue epithelia and at the invasive front of EY carcinoma 20 days after transgene induction. ANOVA with Tukey correction for multiple comparisons: ***p<0.001, ****p<0.0001. (c) Collagenase gene expression across epithelial cell states by scRNAseq. (d) Percent of CD45^+^ cells present in tongue epithelia at 1, 6, 10, and 15 days after transgene induction by flow cytometry. Each time point was analyzed individually by ANOVA with Tukey correction for multiple comparisons: **p<0.01, ****p<0.0001. (e) UMAP of immune cell clusters identified by scRNAseq, n=11,286 immune cells. (f) Immune cell types stratified by transgenic condition. (g) Distribution of immune cell types in each transgenic condition. (h) Expression of chemokine genes (ligands) among epithelial cell clusters. (i) Expression of receptor genes among immune cell clusters for corresponding ligands in h. (j) Representative immunofluorescence image of pan-Cytokeratin, LY6G, and IBA1 expression in N, E, Y, and EY tongue epithelia 20 days after transgene induction. (k) MMP gene expression across immune cell types. (l) Experimental approach for depletion of LY6G^+^ G-MDSCs in transgene induced EY mice. (m) Number of infiltrative carcinoma lesions per examined tongue upon treatment in control mice and mice treated with anti-LY6G depleting antibody.

The basement membrane represents an anatomic barrier against invasive carcinoma. Second harmonic generation microscopy^46^ identified a dramatic reduction in fibrillar collagen abundance at the invasive front of nascent infiltrative carcinoma (Fig. 6a,b). However, CSCs did not express collagenases (Fig. 6c), and hence may lack the intrinsic ability to initiate tumor invasion. In search for an alternative mechanism, we hypothesized that infiltrating immune cells (CD45^+^) observed within EY lesions (Fig. 6d) may contribute to basement membrane extracellular matrix (ECM) remodeling. To explore this possibility, we analyzed the scRNAseq data of 11,286 immune cells present in our scRNAseq data, which distributed across 13 cluster identities (Fig. 6e and Extended Data Fig. 6a,b, Supplementary Table 5). Remarkably, myeloid derived suppressor cells (MDSCs) comprised 65% of immune cells (31% of granulocytic, G-MDSCs, and 34% of monocytic, M-MDSCs) in EY-expressing epithelia (Fig. 6f,g). Ligand-receptor analysis revealed that CSCs express chemokine ligands (*Cxcl1, Cxcl2, S100a8, S100a9, Csf3*) whose corresponding receptors (*Cxcr2, Csf3r*) are specifically expressed by G-MDSCs (Fig. 6h,i). Analysis of bulk RNAseq from epithelia also demonstrated granulocyte genes including *Csf3, Retnlg, Cd177,* and *Ly6g* ranked among the most highly upregulated genes in EY epithelia (Extended Data Fig. 6c). In oral epithelia, TNFα, G-CSF, IL-23, and IL-17 initiate a cytokine-chemokine cross talk, which induces sustained granulocyte recruitment during inflammation.^47^ In line with this model, EY-expressing epithelia showed increased abundance of *Il23a* and *Il17f* transcripts and TNFα, G-CSF, and IL-17 proteins, as well as the granulocyte-specific chemokines CXCL1 and CXCL2 (Extended Data Fig. 6d).

These findings suggest that promoting G-MDSC infiltration constitutes a distinctive feature of CSCs. We thus asked if G-MDSCs contribute to the pre-invasive to invasive transition of CSCs. We observed increased abundance of LY6G^+^ G-MDSCs at tumor invasive fronts (Fig. 6j). Furthermore, in contrast to the absence of collagenase expression in EY epithelial cells, G-MDSCs expressed collagenases *Mmp8* and *Mmp9* (Fig. 6k), and displayed elevated levels of pro-MMP9 protein (Extended Data Fig. 6e). Consistent with a causal role of G-MDSCs in tumor invasion, treatment of transgene-induced EY mice with anti-LY6G depleting antibody significantly reduced the overall multiplicity of carcinoma (Fig. 6l,m and Extended Data Fig. 6f). These findings suggest that cytokines and chemokines secreted by EY-transformed CSCs recruit collagenase-producing G-MDSCs, which cleave basement membrane collagen fibrils and facilitate tumor invasion.

### CSC programs are enriched in HNSC and associated with poor prognosis

**Figure 7:**
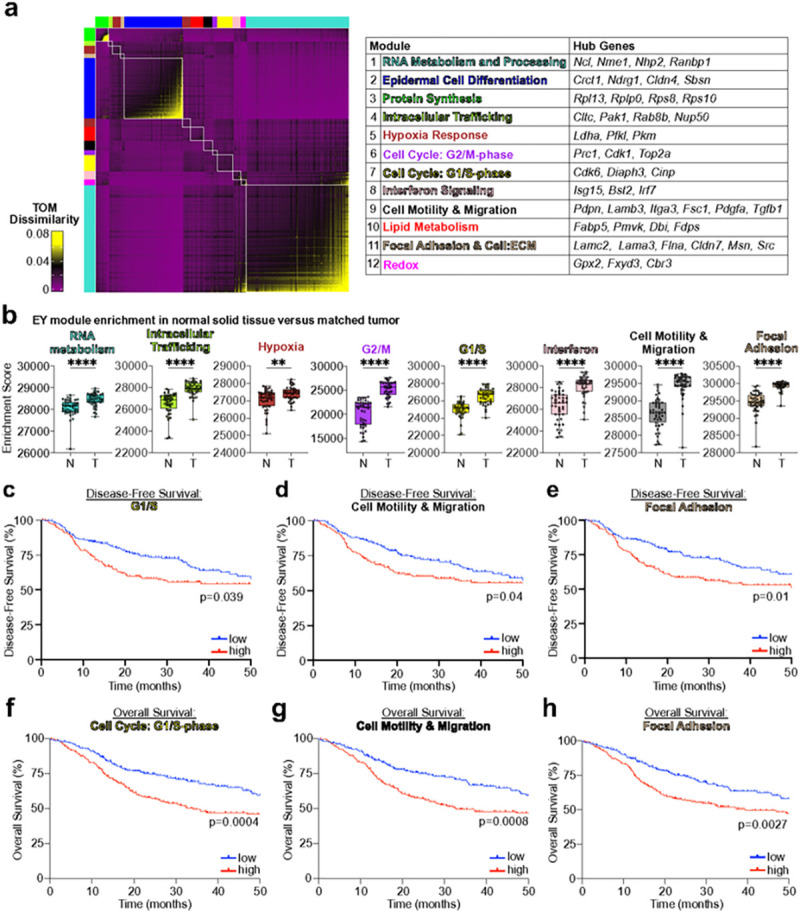
CSC programs are enriched in HNSC and associated with poor prognosis (a) Left: Weighted gene co-expression network analysis (WGCNA) heatmap displaying topological overlap matrix (TOM) dissimilarity indices among genes in TP cluster cells. Right: Table of WGCNA modules and selected genes identified by Metascape and Enrichr. See also Supplementary Table 6. (b) Module enrichment in malignant tumors (T) compared to matched adjacent normal solid tissues (N) by single sample GSEA among TCGA-HNSC subjects (n=43 subjects with matched T and N samples). Two-tailed paired T-test: **p<0.01, ****p<0.0001. (c-e) Kaplan-Meier plots demonstrating worse disease-free survival (n=393) among TCGA-HNSC subjects with greater median enrichment for the (c) G1/S, (d) Cell Motility & Migration, and (e) Focal Adhesion EY-modules. (f-h) Kaplan-Meier plots demonstrating worse overall survival (n=520) among TCGA-HNSC subjects with greater median enrichment for the (f) G1/S, (g) Cell Motility & Migration, and (h) Focal Adhesion modules.

To test the translational significance of the CSC tumor initiation transcriptional program in human disease, we performed high dimensional weighted gene co-expression network analysis (hdWGCNA)^48,49^. We decomposed the tumor initiating cluster into 12 co-expressed transcriptional modules (Fig. 7a, Supplementary Table 6), then subjected each module to ssGSEA analysis in matched TCGA-HNSC tumor and normal tissue samples. We noted enrichment of 8 of 12 modules exclusively in HNSC, unveiling RNA metabolism and processing, intracellular trafficking, hypoxia response, G1/S and G2/M cell cycle progression, interferon response, motility and migration, and cytoskeleton and cell polarity as distinguishing features of the HNSC transcriptome (Fig. 7b). Among all modules, enrichment for CSC G1/S cell cycle, motility and migration, and cytoskeleton and polarity modules were associated with worse disease-free and overall survival (Fig. 7c–h). These findings suggest that our transgene-induced oral CSCs display coherent transcriptional programs enriched in aggressive HNSC.

## DISCUSSION

The overwhelming majority of work investigating cancer-driving mechanisms has relied on established tumors, which requires retrospective inference of tumor initiating events and limits distinction between processes governing tumor initiation from progression. In HNSC, despite this mutational and cellular heterogeneity, the diverse genomic alterations observed in HNSC converge to activate a limited number of oncogenic signaling pathways. Through the use of a spatiotemporally controlled murine system targeting genomic alterations to a single pool of epithelial progenitor cells, we now show that unrestrained YAP activation in the context of *TP53* and *CDKN2A* inhibition by HPV E6-E7 oncogenes induces carcinoma with rapid kinetics and nearly complete penetrance, enabling in-depth investigation of tumor initiation. The ability to combine this genetically-defined system with lineage tracing and single cell transcriptomics provided a unique opportunity to investigate the conversion of epithelial progenitor cells into CSC as it occurs *in vivo*.

The identification of a rapidly emerging CSC population *in vivo* offered the opportunity to directly examine the biological processes giving rise to cancer initiating cells. In this regard a widely accepted framework for carcinogenesis posits that accumulating genomic alterations lead to stepwise transcriptional changes driving normal tissue to invasive carcinoma via a series of intermediate states. Our findings indicate that once a minimal complement of pathway alterations is achieved – loss of *TP53* and *CDKN2A* function and YAP activation – OEPCs are rapidly reprogrammed into CSCs endowed with hallmarks of invasive carcinoma, including cell cycle progression, hypoxia, squamous differentiation defects, interferon responses, and a pEMT cell identity. While the role of pEMT in tumor initiation has remained elusive, our interrogation of the CSC state now reveals that pEMT represents an early and distinguishing phenotype of CSCs. By examining coordinately expressed programs in CSCs, we further dissected the elements of pEMT relevant to transgene-mediated tumor initiation into distinct transcriptional programs driving cellular motility and cell-ECM interactions. Remarkably, our new CSC-specific transcriptional programs, including pEMT, were enriched in HNSC and correlated with worse prognosis, thus supporting their likely direct relevance to human squamous malignancies.

One unexpected finding was that tumor initiation could be achieved in the absence of genomic alterations in the PI3K/AKT/mTOR signaling axis, given the extensive evidence implicating widespread mTOR activation in HNSC^28,50–52^. However, we observed robust mTOR activation in pre-invasive lesions and throughout SCC in YAP-expressing epithelia, suggesting that YAP can activate mTOR signaling. Indeed, mTOR program enrichment represented a distinguishing feature of the EY-induced CSC state. Mechanistically, we found that YAP drives the transcriptional upregulation of *Axl* and multiple EGFR ligands, which may explain EY-mediated mTOR activation. The fact that > 70% of HNSCs do not harbor genomic alterations in the PI3K-mTOR pathway but exhibit a widespread activation of YAP^17,53^ and mTOR^52^ is aligned with a potential signaling cross-talk in which YAP can activate mTOR. Specifically, our findings support a model in which YAP:TEAD-mediated transcription activates an AXL- and EGFR-initiated signaling cascade resulting in the activation of mTOR in epithelial cells, thus representing an actionable target to prevent tumor initiation.

Remarkably, transgene-induced carcinoma displayed tumor invasive fronts with extensive modification of basement membrane collagen fibrils, concordant with the overall perspective that ECM modification and invasion are defining features of premalignancy-to-cancer transition. However, our single cell analysis of CSCs revealed that these cells do not express collagenases. Instead, our findings indicated that CSCs express multiple cytokines and chemokines that promote G-MDSC recruitment, and that recruitment of G-MDSCs expressing *Mmp8* and *Mmp9* collagenases to the invasive front is required for CSC invasion. These observations provide a model in which CSCs lack cell autonomous intrinsic invasive capacity and alternatively polarize the tumor microenvironment (TME) and exploit innate immune cells to disrupt the basement membrane, ultimately enabling tumor invasion.

**Figure 8:**
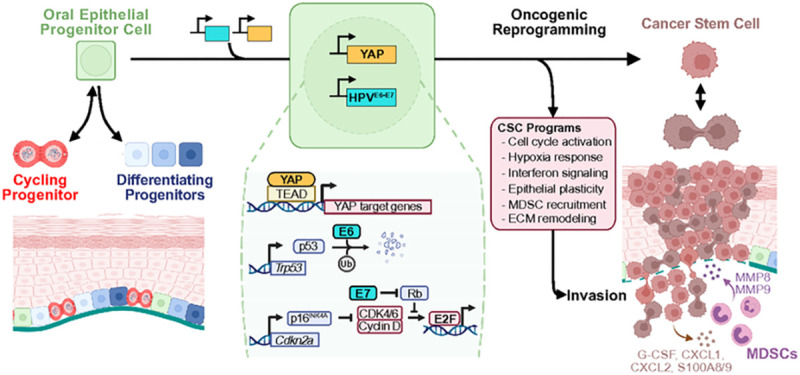
Genetically-defined oncogenic and tumor suppressive pathway alteration in normal oral epithelial progenitor cells defines tumor initiating events.

In summary, we demonstrate that a genetically-defined, traceable system simultaneously activating oncogenic pathways and disabling tumor suppressive mechanisms in normal oral epithelial progenitor cells induces the emergence of a distinct cancer initiating stem-like cell state. Through multimodal analysis of nascent CSCs at the single cell level *in vivo*, we define tumor-autonomous transcriptional programs and CSC-TME cross-talk as tumor initiating events during invasive carcinoma formation (Fig. 8). This conceptual framework of cancer initiation has the potential to open multiple novel avenues for early intervention, including precision targeting of tumor cell-autonomous cancer initiating signaling pathways, and disrupting CSC-TME networks mediating the development of invasive carcinoma.

## RESOURCE AVAILABILITY

### Lead contact

Further information and requests for resources and reagents should be directed to the Lead Contact, J. Silvio Gutkind (sgutkind@health.ucsd.edu).

### Materials availability

Transgenic mice and cell lines generated in this study are available from the Lead Contact upon reasonable request and completion of Material Transfer Agreements (MTA). There may restrictions to the availability of these reagents due to cost or limited quantities.

## ONLINE METHODS

### Genotyping PCR primers

**Table:** 

Oligo name	Sequence	Description	Amplicon Size
K14CreERT_F	CGCATCCCTTTCCAATTTAC		169bp
K14CreERT_R	GGGTCCATGGTGATACAAGG		
Col1_wt_F	tccctcacttctcatccagatatt		
Col1_wt_R	agtcttggatactccgtgaccata	wt:	1092 bp
Col_mut_R	ggacaggataagtatgacatcatcaa	mutant:	480 bp (*YAP1^S127A^*)
Trp53_F	cttggagacatagccacactg	Internal Control	
Trp53_R	ttacacatccagcctctgtgg		166 bp
Il2_F	CTAGGCCACAGAATTGAAAGATCT	Internal Control	324 bp
Il2_R	GTAGGTGGAAATTCTAGCATCATCC		
HPV16-E67_F2	CTGAGAACAGATGGGGCACA		201 bp
HPV16-E67_R2	GACAGCTCAGAGGAGGAGGA		
rtTA_F	TGCCAACAAGGTTTTTCACTAGAGA		90 bp
rtTA_-R	CTCTTGATCTTCCAATACGCAACCTA		
H2B-GFP-F	GCTCGTTTAGTGAACCGTCAG		250 bp
H2B-GFP-R	GACTGTGTCTGATTTCC		
Ctrl_F	AGTGGCCTCTTCCAGAAATGTGC	Internal Control	521 bp
Ctrl_R	TCTTCTGCGCCTTAGTCACC		

### Loxp–STOP–Loxp excision assay PCR primers

**Table:** 

Oligo name	Sequence	Amplicon Size	Description
LSL_link_pA_F1	gcctgaagaacgagatcagc	1.5 kb	Intact
LSL_link_recomb_F1	caaactcttcgcggtctttc	400 bp	Recombined
LSL_link_rtTA_R2	AAAATCTTGCCAGCTTTCCCC		

### RT-qPCR primers

**Table:** 

Oligo name	Sequence	Amplicon Size	Description
hsaYAP1_qPCR_F1	TAGCCCTGCGTAGCCAGTTA	177 bp	Transgene specific Tang et al.^54^
hsaYAP1_qPCR_R1	TCATGCTTAGTCCACTGTCTGT		
HPV16_E6_qPCR_F1	AATGTTTCAGGACCCACAGG	107 bp	Bordigoni et al.^55^
HPV16_E6_qPCR_R1	GTTGCTTGCAGTACACACATTC		
HPV16_E7_qPCR_F1	TCAGAGGAGGAGGATGAAATAGA	111 bp	
HPV16_E7_qPCR_R1	GCACAACCGAAGCGTAGA		
mmuPpia_qPCR_F1	GAGCTGTTTGCAGACAAAGTTC	125 bp	PrimerBank validated^56^
mmuPpia_qPCR_R1	CCCTGGCACATGAATCCTGG		

### IHC antibodies

**Table:** 

Primary Antibody	Clone	Dilution	Species	Supplier
Pan-cytokeratin	ab9377	1/200	Rabbit	Abcam
phospho-S6	CST2211	1/400	Rabbit	Cell Signaling Technology
KI67	ab15580	1/400	Rabbit	Abeam
P63	CST39692	1/900	Rabbit	Cell Signaling Technology
SOX2	CST14962	1/300	Rabbit	Cell Signaling Technology

**Table:** 

Secondary Antibody	Dilution	Catalog #	Supplier
Goat Anti-Rabbit IgG Antibody (H+L), Biotinylated	1/200	BA-1000	Vector Laboratories

### Immunofluorescence antibodies

**Table:** 

Primary Antibody	Clone	Dilution	Species	Supplier	Secondary Antibody	Secondary dilution	Secondary catalog #
KRT14	poly19053	1/200	Rabbit	BioLegend	AF568 goat anti-rabbit	1/1000	Thermo A11036
PDPN-biotin	8.1.1	1/100	Syrian Hamster	BioLegend	AF647 streptavidin	1/1000	Thermo A78962
KRT15	Poly18339	1/100	Chicken	BioLegend	AF674 goat anti-chicken	1/1000	Thermo A11036
KI67	ab15580 (poly)	1/200	Rabbit	Abeam	AF568 goat anti-rabbit	1/1000	Thermo A32933
ITGA6	GoH3	1/200	Rat	BioLegend	AF647 goat-anti-rat	1/1000	Thermo A21247
P63	D9L7L	1/200	Rabbit	CST	AF568 goat anti-rabbit	1/1000	Thermo A32933
IBA1	E404W	1/200	Rab	CST	AF568 goat anti-rabbit	1/1000	Thermo A32933
LY6G	1A8	1/100	Rat	BioLegend	AF647 goat-anti-rat	1/1000	Thermo A21247
Broad Spectrum Cytokeratin	poly ab86734	1/200	Mouse	Abcam	AF488 goat-anti-mouse	1/1000	Thermo A21121

### siRNAs

**Table:** 

Name	Description	Supplier	Catalog number	Sequence
siCTRL	Non-targeting Pool	Horizon Discovery	D-001810-10-20	UGGUUUACAUGUCGACUAA
				UGGUUUACAUGUUGUGUGA
				UGGUUUACAUGUUUUCUGA
				UGGUUUACAUGUUUUCCUA
siYAP	Human YAP1 Pool	Horizon Discovery	L-012200-00-0005	GCACCUAUCACUCUCGAGA
				UGAGAACAAUGACGACCAA
				GGUCAGAGAUACUUCUUAA
				CCACCAAGCUAGAUAAAGA
siTAZ	Human WWTR1 Pool	Horizon Discovery	L-016083-00-0005	CCGCAGGGCUCAUGAGUAU
				GGACAAACACCCAUGAACA
				AGGAACAAACGUUGACUUA
				CCAAAUCUCGUGAUGAAUC
siAXL	Human AXL Pool	Horizon Discovery	L-003104-00-0005	ACAGCGAGAUUUAUGACUA
				GGUACCGGCUGGCGUAUCA
				GACGAAAUCCUCUAUGUCA
				GAAGGAGACCCUUAUGGA

### Immunoblot antibodies

**Table:** 

Primary Antibody	Clone	Dilution	Species	Supplier
YAP/TAZ	D24E4	1/1000	Rabbit	Cell Signaling Technology
AXL	C89E7	1/1000	Rabbit	Cell Signaling Technology
CYR61	D4H5D	1/1000	Rabbit	Cell Signaling Technology
pEGFR	1H123	1/1000	Mouse	Cell Signaling Technology
EGFR	D38B1	1/1000	Rabbit	Cell Signaling Technology
pS6	D68F8	1/1000	Rabbit	Cell Signaling Technology
S6	54D2	1/1000	Mouse	Cell Signaling Technology

**Table:** 

Secondary Antibody	Dilution	Catalog #	Supplier
HRP-goat anti-rabbit IgG	1/10,000	4030-05	Southern Biotechnology
HRP-goat anti-mouse IgG	1/10,000	1030-05	Southern Biotechnology

## EXPERIMENTAL MODEL AND SUBJECT DETAILS

### Mouse lines

Mice were house in accordance with University of California San Diego Institutional Animal Care and Use Committee (IACUC) guidelines. The UCSD IACUC approved all mouse experiments (Protocol S15195). The following mouse lines were kindly provided by Dr. Elaine Fuchs (The Rockefeller University): Tg(KRT14-cre/ERT)^20Efu^ and Tg(tetO-HIST1H2BJ/GFP)^47Efu^.^19,20^ The Col1a1tm1(tetO-Yap1*)^Lrsn^ mouse was kindly provided by Dr. Fernando Camargo (Harvard University).^21^ The B6.Cg-Gt(ROSA)26Sor^tm1(rtTA,EGFP)Nagy/J^ mouse was obtained from The Jackson Laboratory.^57^ The Tg(tetO-HPV16-E6E7)^SGu^ mouse was designed by the Gutkind laboratory and generated in house.^16^ All transgenic mouse experiments were performed in age- and sex-balanced groups of 8–16 week old littermates. NSG^™^ mice (NOD.Cg-*Prkdc*^*scid*^ *Il2rg*^*tm1Wjl*/SzJ^) mice were originally obtained from The Jackson Laboratory and propagated at the Moores Cancer Center. Implantation of transgene epithelial cell suspensions were performed in 8-week-old female NSG mice.

### Human HNSC cell lines

CAL27 and CAL33 cell lines were obtained from the NIDCR Oral and Pharyngeal Cancer Branch cell collection.^58^ Cell identity was confirmed by STR profiling. CAL27 (CVCL_1107) was derived from a 56 year old male with tongue adenosquamous carcinoma. CAL33 (CVCL_1108) was derived from a 69 year old male with tongue squamous cell carcinoma. Both cell lines were cultured in DMEM (D-6429, Sigma-Aldrich, St. Louis, MO), 10% fetal bovine serum, 5% CO_2_, at 37 °C, and both tested free of Mycoplasma infection directly prior to experimentation.

### TCGA-HNSC

Transcriptome profiling, biospecimens, and clinical data from The Cancer Genome Atlas Program (TCGA) was downloaded from the National Cancer Institute GDC Data Portal for patients with the cancer type head and neck squamous cell carcinoma (HNSC) (https://portal.gdc.cancer.gov/projects/TCGA-HNSC)]^14^ Additional clinical data for this project was downloaded from cBioPortal (https://www.cbioportal.org/study/summary?id=hnsc_tcga_pan_can_atlas_2018).^59,60^

## METHOD DETAILS

### Husbandry and genotyping

We bred mice expressing E6-E7 (*Krt14-CreERT/LSL-rtTA/tetON_H2B-GFP/tetON_E6-E7*, “**E**”), *YAP1*^*S127A*^ (*Krt14-CreERT/LSL-rtTA/tetON_H2B-GFP/tetON_YAP1*^*S127A*^, “**Y**”), or both transgenes (*Krt14-CreERT/LSL-rtTA/tetON_H2B-GFP/tetON_E6-E7/tetON_YAP1*^*S127A*^, “**EY**”). Littermates that bore neither *tetON_E6-E7* nor *tetON_YAP1*^*S127A*^ effector transgenes but possessed the *Krt14-CreERT* and *LSL-rtTA* regulatory transgenes were used as the normal condition (*Krt14-CreERT/LSL-rtTA/tetON_H2B-GFP*, “**N**”). Intralingual injection of tamoxifen was performed to achieve reliable transgene induction. Mice were started on a doxycycline-containing diet on the first day of tamoxifen treatment. This treatment regimen resulted in consistent CreERT-mediated excision of the floxed STOP cassette and expression of effector and reporter transgenes in KRT14^+^ basal cells.

*Krt14-CreERT*^*+/+*^*/LSL-rtTA*^*+/+*^*/H2B-GFP*^*+/+*^*/E6-E7*^*+/−*^ mice were crossed to *Krt14-CreERT*^*+/+*^*/LSL-rtTA*^*+/+*^*/H2B-GFP*^*+/+*^*/YAP1*^*S127A+/−*^ resulting in Mendelian proportions of N, E, Y, and EY littermates. At 3–4 weeks of age, a tail fragment was obtained for initial screening genotype confirmation. Directly prior to transgene induction for experiments, mice were assigned to age and sex-balanced groups, and an ear fragment was obtained for confirmatory genotyping. Genomic DNA was isolated by incubating tissue in 25mM NaOH and 0.2 mM EDTA at 100°C for 1 hour, followed by neutralization with an equal volume of 40 mM Tris-HCl (pH 5.5).^61^ Multiplex polymerase chain reaction (PCR)-based genotyping was performed using REDTaq^®^ polymerase per manufacturer recommendations (Millipore Sigma). Oligonucleotides were multiplexed as follows: (1) *LSL-rtTA* and *E6-E7* and *Il2* (positive control), (2) *Yap1*^*S127A*^ and *Trp53* (positive control), (3) *Krt14-CreERT* and *Il2* (positive control). All PCR products were subjected to electrophoresis on 2% agarose gel in Tris acetate EDTA buffer.

### Transgene induction

Mice were anesthetized with isoflurane and 100μL of tamoxifen solution (20 mg/mL in miglyol) was administered into the tongue under stereomicroscopic visualization. One dose of tamoxifen was administered every other day for a total of 3 doses.

### Epithelia isolation

After *in situ* infiltration with 500uL collagenase+dispase solution (1mg/mL, 2.5 mg/mL) (Millipore Sigma), the tongues of euthanized mice were dissected free and incubated for 30 minutes at 37°C. The tongue epithelium was then dissected free from the underlying muscle under stereomicroscopic visualization.

### LoxP–STOP–LoxP excision assay

For floxed stop cassette excision assay, high-quality genomic DNA was isolated from whole epithelia using the DNeasy Blood and Tissue Kit per manufacturer protocol (Qiagen). PCR products were generated using REDTaq^®^ polymerase and LSL excision primers, and were subjected to electrophoresis on 2% agarose gel in Tris acetate EDTA buffer.

### RT-qPCR

RNA was prepared by homogenization of whole tongue epithelia in TRIzol^®^ (Invitrogen) followed by phenol:chloroform extraction and RNeasy Mini Kt based column purification with on-column DNase treatment (Qiagen). For quantitative PCR (qPCR), cDNA library preparation was performed using Bio-Rad iScript^™^ reverse transcriptase and qPCR was performed using Applied Biosystems Fast SYBR^®^ Green Master Mix per manufacturer’s instructions.

### Evaluation of gross tongue lesions

Following transgene induction, mice were examined under anesthesia using a stereomicroscope every 3–7 days for the appearance of tongue epithelial lesions. Lesion free survival in days was defined as the time from first tamoxifen treatment to the appearance of the first gross lesion.

### Histopathology and immunohistochemical staining

Mouse tongues were transected from the pharynx and floor of mouth, and placed in 10% aqueous buffered zinc formalin for 24–36 hours at room temperature and transferred to 70% ethanol. Tissues were paraffin embedded, sectioned (5μm), and stained with hematoxylin and eosin (H&E) by standard protocols (Histoserv or LJI histology). H&E slides were prepared according to a standard protocol. https://www.protocols.io/view/hematoxylin-amp-eosin-protocol-for-leica-st5020-au-x54v9mozqg3e/v1

Immunohistochemistry (IHC) was performed as previously described.^62^ After deparaffinization, antigen retrieval was performed using IHC Antigen Retrieval Solution (ThermoFisher, 00–4955-58) in a steamer for 40 minutes. Endogenous peroxidases were inactivated using Bloxall Blocking Solution (Vector Labs, SP-6000, 30-min incubation, room temperature). Tissues were incubated with primary antibody overnight at 4°C then exposed to biotinylated anti-rabbit secondary antibody (Vector Labs, BA-1000, 1:400 dilution, 30 min at room temperature followed by avidin-biotin complex formation (Vector Laboratories, # PK-6100), staining with DAB substrate (Vector Laboratories, # SK-4105), and hematoxylin counterstain (Mayer’s Hematoxylin solution (Sigma MHS1–100ML)). All H&E and IHC stained slides were scanned using the Leica Aperio AT2 slide scanner at 40x magnification.

### Fluorescence microscopy

For whole mount fluorescence imaging, epithelial sheets were isolated from mice euthanized 36 hours after a single dose of intralingual tamoxifen, washed in HBSS, stained with NucBlue for 3 hours at room temperature, washed again, mounted immersed in HBSS between two cover glasses, and Z-stacks were acquired with a confocal microscope.

For cross-sectional fluorescence imaging, immediately after euthanasia, mice underwent intracardiac perfusion first with 2mM EDTA in PBS followed by 1.6% paraformaldehyde in PBS. Perfusion fixed tongues were dissected and incubated in 1.6% paraformaldehyde at room temperature overnight, then transferred to 30% sucrose in PBS for 2–3 days at 4°C, then washed in PBS, then embedded in OCT media and snap frozen in cryomolds for frozen section slide preparation. For fluorescent analyses, slides were thawed in the dark, blocked, incubated overnight at 4°C with primary antibodies, and then incubated with fluorophore-conjugated secondary antibodies for 2 hours at room temperature. Nuclei were then stained with Hoechst 33342 in PBS for 15 minutes and slides were mounted with ProLong Diamond mounting medium.

### Second harmonic generation for collagen imaging

The second-harmonic generation imaging was done on an upright Leica SP8 microscope with a resonant scanner and hybrid non-descanned detectors. Ti-Sapphire femtosecond pulsed Chameleon Ultra II (Coherent Inc.) laser was tuned to 855 nm and the beam was focused on the sample with an HC PL APO CS 10x/0.40 dry objective. The light was routed to the detectors with 560 nm, 495 nm, and 640 nm long-pass dichroic mirrors. The SHG signal was recorded with a 425/26 nm bandpass filter, the autofluorescence was recorded with a 650/60 nm bandpass filter. The pixel size was set to 0.746 μm, and 16x line averaging was used to improve the signal-to-noise ratio. Data were digitized in an 8-bit mode. The sample navigator software module was used to create autofocus support points and individual fields of view were tiled and stitched.

### RNAseq

Tongue epithelia were isolated and RNA was prepared as described above. RNA samples passing purity, concentration, and integrity quality metrics by NanoDrop and TapeStation were submitted to Novogene for oligo-dT-based mRNA selection, cDNA library preparation, and sequencing on Illumina NovaSeq6000.

### siRNA transfection in human cell lines

All human cells were transfected at 60% confluency using Lipofectamine RNAiMAX reagent according to the manufacturer’s instructions, using 20nM of each siRNA. Culture media was refreshed at 24 hours after transfection. Cells were placed under serum free conditions at 48 hours, and collected for experimentation at 72 hours post-transfection.

### Immunoblot assay

Cells rinsed with ice cold PBS and lysates were harvested in RIPA buffer (50 mM Tris-HCl, 150 mM NaCl, 1 mM EDTA, 1% NP-40) supplemented with Halt^™^ Protease and Phosphatase Inhibitor Cocktail (#78440, ThermoFisher Scientific) and cleared by centrifugation for 15 minutes. The concentration of supernatants was measured using Bradford colorimetric assay. Equal amounts of protein were loaded onto 10% polyacrylamide gels, subjected to electrophoresis in Tris/Glycine/SDS buffer, and transferred to PVDF membranes. The membranes were blocked with 5% milk in TBS with 0.1% Tween-20 (TBS-T) buffer for 1 hour, incubated with primary antibodies diluted in 5% BSA overnight at 4°C. After washing 3 times with TBS-T, the membranes were incubated with HRP-conjugated secondary antibodies diluted in 5% milk in TBS-T for 1 h at room temperature. Immobilon Western Chemiluminescent HRP substrate (Millipore, MA) was used for detection.

### Cytokine array

Whole epithelia were homogenized in RIPA lysis buffer with protease and phosphatase inhibitors, snap frozen, and sent to Eve Technologies for Mouse Cytokine/Chemokine 44-Plex and Mouse MMP 5-plex Discovery Assay^®^ Arrays.

### Generation of epithelial cell suspensions

Isolated epithelia were minced in 0.25% trypsin-EDTA (Thermo) and subjected to mechanical dissociation in the gentleMACS dissociator C tubes (Miltenyi #130–095-937) for 12 minutes at 37°C, followed by inactivation of trypsin and filtration.

### Primary epithelial cell culture

Mouse tongue epithelial cells were isolated from mice following transgene induction as described above. Cells were grown on collagen coated plates in complete DermaCult keratinocyte basal expansion medium (STEMCELL Technologies). Medium contained the manufacturer’s provided supplements, plus 5 ng/mL mouse EGF (Gibco), 50 pM cholera toxin (Sigma), 1x antibiotic/antimycotic solution [Gibco], and 2 uM doxycycline hyclate (Sigma, to maintain transgene activation) at 37°C with 5% CO_2_.

### Flow cytometry

Epithelial cell suspensions were stained for viability using LIVE/DEAD^™^ Fixable Blue Dead Cell Stain Kit (Thermo #L23105) and BUV737 Rat Anti-Mouse CD45 Clone 30-F11 (BD Biosciences # 568344) and analyzed using a 5-laser Cytek Aurora.

### Cell sorting

EY epithelia were isolated and maintained in culture as previously above. When the cells were approximately 70–80% confluent, single cell suspensions were generated by subjecting the cells to EDTA and then trypsin, and then mechanically lifting the cells. Cells were counted and viability assessed by trypan blue staining using a Countess III cell counter. The cells were then resuspended in HBSS at a concentration of ~10 million cells/mL and subjected to fluorescence activated cell sorting (FACS) using an Aria II cell sorter. Single cells were identified based on forward and side scatter parameters, and then GFP positive and negative cells were sorted into individual tubes with cell culture medium. Sorted cells were returned to culture and expanded for experimentation and cryopreservation.

### scRNAseq

Single cell suspensions were generated from tongue epithelia, sorted for viability, and subjected to droplet-based single cell cDNA library preparation and sequencing. Two mice per genotype were used to generate tongue epithelial cell suspensions. Cells were sorted on a BD FACSAria-II. Viable single cells were selected by size (FSC x SSC) and viability (double negative for propridium iodide and Fixable Viability Dye eFluor780 (eBioscience) staining) parameters. Sorted cells were then loaded on a Chromium Controller (10x Genomics) using the Chromium Next GEM Single Cell 3’ Kit v3.1 (10x #1000269) according to the manufacturer’s protocol with a target of 10,000 cells per GEM reaction. The resulting cDNA library was sequenced on an Illumina NovaSeq 6000 using the S1 100 cycle Reagent Kit v1.5 (Illumina 200228319), with a targeted read depth of 20,000 reads/cell.

### Orthotopic implantation

Epithelial cell suspensions were generated as described above. Cell count and viability was performed using trypan blue on the Countess III. Cells were only implanted for epithelia that showed minimum viability of 75%. After one wash in HBSS, 2×10^5^ viable cells were implanted orthotopically into the tongues of NSG mice. After implantation, mice tongues were first evaluated at 5 days after implantation then every other day until endpoint was reached.

### Imaging equipment and software

Gross evaluation of tongue lesions (Fig. 1 & 5) was performed using the Motic K-400P stereo microscope. Fluorescent cross-sectional images of were acquired using the Zeiss LSM780 confocal microscope system with Zeiss Black software (Fig. 2a, S1, S2, 6), Zeiss AxioZ1 slide scanner (Fig. 2c), or Zeiss LSM990 confocal microscope system with Zeiss Blue software (Extended Data Fig. 5). Histological images (H&E, IHC, immunofluorescence) were analyzed using QuPath 0.2.3, ImageJ/FIJI, or MATLAB.

## QUANTIFICATION AND STATISTICAL ANALYSIS

### Histopathological analyses

Histopathological changes for each experimental condition were independently evaluated by at least two board-certified veterinary pathologists (KK, KSakaguchi, AAM). Carcinoma was defined as atypical epithelial cells deep to the basement membrane. Average epithelial thickness was determined on mid-tongue axial sections by measuring 8–10 orthogonal lines from the basement membrane to the epithelial surface. Carcinoma burden was defined as the number of independent carcinoma foci identified in individual tongues. Carcinoma size was defined as the cross-sectional area of atypical epithelial cells invading deep to the basement membrane. Carcinoma depth of invasion was measured using a line orthogonal to the basement membrane of the closest adjacent normal mucosa to the deepest point of tumor invasion.

### Quantification of IHC

Basal phospho-S6 staining was quantified in QuPath using a trained pixel classifier applied to manually-segmented epithelial basal layer regions of interest (ROI). At least three ROIs each with a minimum area of 20,000 um^2^ were analyzed per sample. The fraction of phosphoS6 positive pixels for each sample was calculated using the mean of the ROIs after adjusting for relative area per ROI. Suprabasal Ki67, p63, Sox2+ nuclei were quantified in a similar fashion using a trained object classifier applied to manually-segmented epithelial subrabasal layer regions of interest. At least three suprabasal layer ROIs with minimum area of 50,000 um^2^ were selected per sample.The fraction of Ki67, p63, or Sox2+ positive nuclei for each sample was calculated using the mean of the ROIs after adjusting for relative area per ROI.

### IF nuclear segmentation

Instance segmentation was performed using Stardist, a deep-learning based segmentation FIJI plugin. Distinct grayscales values were assigned to each nucleus called by Stardist. MATLAB scripts were then developed to parse the label images, placing the linear indices corresponding to each pixel within a nucleus into a cell array. The relative size of each cell array corresponded to the number of segmented nuclei per image. The Hoechst and GFP channels from each confocal image were separated for independent segmentation and cell array formation. The ratio of GFP+ to Hoechst+ nuclei were calculated by comparing the resulting nuclear pixel cell arrays for the GFP and Hoechst channels for each image. The Hoechst channel was used for normalization and calculation of the mean fluorescence intensity (MFI) in the GFP channel. A threshold value was calculated using this normalized MFI to call Hoechst+/GFP+ and Hoechst+/GFP− nuclei.

### IF spatial context analysis

To determine the spatial fluorescent intensity distribution of ITGA6 as a function of distance from the basement membrane, MFI was calculated along the manually traced basement membrane by calculating vertical shifts from the basement membrane on a per-pixel basis. An array of each pixel location was created, and the MFI along the length of the traced basement membrane was calculated. As epithelial intensity distributions varied based on the length of the basement membrane tracing, they were interpolated using a spline with 50,000 query points, allowing the arrays to be combined and a normalized average intensity distribution to be calculated for each set of image arrays for a given mouse.

### Statistical analyses

Statistical analyses were performed in GraphPad Prism 9.5.1 with an alpha threshold of 0.05. Groupwise comparisons were tested using Kruskal-Wallis (one-way ANOVA on ranks) test with Dunn’s post-hoc correction for multiple comparison. Differences in survival were compared by Mantel-Cox Log-Rank test with Bonferroni correction for multiple comparisons. Pairwise comparisons between normal and malignant tumors was conducted using two-tailed paired t test. The correlation between mTOR and YAP signatures among TCGA tumors was determined initially by performing a simple linear regression and tested for significance using a two-tailed Spearman’s test. Additional statistical analyses on bulk RNAseq and scRNAseq data were performed in R (v4.1.2, 2021–11-01, “Bird Hippie”); see respective sections for details.

### Bulk RNAseq analyses

Paired-end reads were aligned using STAR v2.7.9 using default settings. STAR index was created using the GRCm39 primary genome FASTA and annotation files. The resulting BAM files were sorted by name using samtools v1.7 then gene counts were quantified using HTSeq-count v0.13.5. Pairwise differential expression was calculated and principal component analysis plots were created using DESeq2 v1.34.0. DEGs were defined at thresholds of p_adj_<0.01 and log_2_FC>1.

### GO and GSEA analyses

Gene ontology (GO) analyses were performed using GeneOntology.org (Panther 17.0) using significantly differentially expressed genes at (|log2FC| > 1, p-value < 0.01). Gene set enrichment analysis (GSEA^63^) was conducted using the Julia packages Match.jl and BioLab.jl, which contain bioinformatics and computational biology functions under active development. Prior to GSEA analyses, raw bulk RNAseq reads were aligned to the human reference transcriptome with the pseudo-aligner Kallisto^64^ using the “quant” command. Transcript expression values were normalized to transcript per million. Transcript expression was converted to gene expression using the maximum individual transcript expression. Single sample GSEA^27^ was performed with rank normalization against MSigDB^33,34^ gene set collections c2, c3, c5, and c6, with 10,000 permutations. Enriched and depleted gene sets were prioritized based on respective information coefficients^65–67^ and Bonferroni-corrected chi-square p-values.

### Global clustering

Single cell gene expression data was processed from the Illumina sequencer files using Cell Ranger (v5.0.0) and its prebuilt mouse reference genome. Individual sample data was then processed and merged using the Seurat (v4.3.0) SCTransform pipeline. Low quality cells (mitochondrial percentage >7, features <1000 and >5500, transcripts per cell >30,000) were filtered prior to data scaling and normalization. After filtering, data was transformed using SCTransform with default parameters, regressing on percent mitochondrial content. Principal component analysis was performed with RunPCA, using the top 50 PCs. Dimensionality reduction was performed with RunUMAP, using the top 30 dimensions. Nearest-neighbor analysis was performed using FindNeighbors using the top 30 dimensions and with k.param set to 50. Clustering was performed with FindClusters with resolution 0.3. Marker genes were calculated using FindAllMarkers with default parameters. Cluster identities were assigned by analysis of differential gene expression.

### Epithelial and immune cell sub-clustering

Epithelial and immune cell subset Seurat objects were generated and analyzed individually using Seurat version 4.3.0. Following assignment of cells to epithelial or immune cell subsets, the analysis pipeline used for the combined analysis was run again for each individual subset with identical parameters. Following initial subset clustering, contaminating residual immune or epithelial cells were removed from the subset Seurat objects, and the subset data reanalyzed in Seurat.

### Transgene alignment

For quantification of the transgene expression in single cells, STARsolo algorithm implemented to STAR aligner version 2.7.9a was applied. Briefly, the custom FASTA file was generated by merging the mm10 mouse genome and the transgene sequences. The index file for this custom genome was generated by STAR using the custom GTF file including the annotations for transgenes with the following parameters; *--sjdbOverhang 100, --genomeSAsparseD 3*. Subsequently, the FASTQ files including cDNA reads and cell barcodes of each sample were aligned to the custom genome by STARsolo with the following parameters; *--soloType CB_UMI_Simple, --clipAdapterType CellRanger4, --outFilterScoreMin 30, --soloCBmatchWLtype 1MM_multi_Nbase_pseudocounts, --soloUMIfiltering MultiGeneUMI_CR, -soloUMIdedup 1MM_CR, --soloCellFilter EmptyDrops_CR*. Finally, the cell-gene count arrays of the transgenes for each sample were obtained as the output of STARsolo. The data was imported to R and normalized with Seurat R package with “NormalizeData” function using the following options; *normalization.method = “LogNormalize”* and *scale.factor = 10000*. The normalized transgene expression arrays were merged with the Seurat object of the epithelial cell cluster by cell barcodes for downstream analysis.

### Weighted Gene Co-expression Network Analysis (WGCNA) of scRNAseq data.

R package hdWGCNA version 0.2.03 (https://smorabit.github.io/hdWGCNA/) was used for WGCNA analysis in the scRNAseq dataset. Normalization of the integrated Seurat object containing cell-gene expression arrays of EY-genotype epithelial cells was performed using NormalizeMetacells using parameters k=10, max_shared=10, min_cells=20. A soft thresholding power was determined as 8 using the function TestSoftPowers and applied for estimation of co-expressing network in the EY-genotype scRNAseq dataset. Significantly co-expressed module genes and highly connected genes within each module (hub genes) were identified by computing eigengene-based connectivity (kME). The heatmap representing topology overlap matrix (TOM) of module genes was generated using R package ComplexHeatmap version 2.14.0. Genes with signed module eigengene-based connectivity measure (kME) greater 0.3 were considered as moderate to high confidence module genes. Modules were assigned functional annotations based on enrichment of member genes for biological processes using Enrichr^68,69^ and MetaScape.^70^

### Sequence: HPV16 ^E6-E7^

Atgcaccaaaagagaactgcaatgtttcaggacccacaggagcgacccagaaagttaccacagttatgcacagagctgcaaacaactatacatgatataatattagaatgtgtgtactgcaagcaacagttactgcgacg

### Sequence: YAP1 ^S127A^ (S127A codon underlined and in bold)

ATGGATCCCGGGCAGCAGCCGCCGCCTCAACCGGCCCCCCAGGGCCAAGGGCAGCCGCCTTCGCAGCCCCCGCAGGGGCAGGGCCCGCCGTCCGGACCCGGGCAACCG
